# Cantharidin induces G_2_/M phase arrest and apoptosis in human gastric cancer SGC-7901 and BGC-823 cells

**DOI:** 10.3892/ol.2014.2611

**Published:** 2014-10-10

**Authors:** CHENJING ZHANG, ZHONGTING CHEN, XINGLU ZHOU, WEN XU, GANG WANG, XIAOXIAO TANG, LAISHENG LUO, JIANGFENG TU, YIMIAO ZHU, WEN HU, XIANG XU, WENSHENG PAN

**Affiliations:** 1 Department of Gastroenterology, The Second Affiliated Hospital of Zhejiang University School of Medicine, Hangzhou, Zhejiang 310009, P.R. China; 2 Department of Pharmacy, The Second Affiliated Hospital of Zhejiang University School of Medicine, Hangzhou, Zhejiang 310009, P.R. China; 3 Department of Gastroenterology, Hangzhou, Zhejiang 310009, P.R. China; 4 Department of Pharmacy, Binjiang Campus of The Second Affiliated Hospital of Zhejiang University School of Medicine, Hangzhou, Zhejiang 310009, P.R. China

**Keywords:** cantharidin, gastric cancer, G_2_/M phase arrest, apoptosis, caspase, Bcl-2 family

## Abstract

The aim of the present study was to investigate the effect of cantharidin (CTD) on human gastric cancer cells and to explore the underlying mechanisms of these effects. The human gastric cancer SGC-7901 and BGC-823 cell lines were treated with CTD. MTS assays were then employed to examine cellular proliferation, flow cytometry was used to analyze the cell cycle and apoptosis, and western blot analysis was used to determine protein expression levels. It was found that CTD inhibited the proliferation of the human gastric cancer SGC-7901 and BGC-823 cells in a dose- and time-dependent manner *in vitro*. CTD also induced G_2_/M phase arrest and cellular apoptosis in a dose-dependent manner. In addition, CTD increased the levels of p21, caspase-7, -8 and -9, activated caspase-3, poly ADP ribose polymerase and Bad, but decreased the levels of cyclin-dependent kinase 1, cyclin A and B, B-cell lymphoma-2 (Bcl-2) and Bid. The present results suggested that CTD may inhibit the proliferation of human gastric cancer SGC-7901 and BGC-823 cells *in vitro* by inducing G_2_/M phase arrest and cell apoptosis. CTD may induce cellular G_2_/M phase arrest by regulating cycle-associated proteins and induce apoptosis by activating a caspase cascade or regulating the Bcl-2 family proteins.

## Introduction

Gastric cancer is the fourth most prevalent malignant disease and the second leading cause of cancer-related mortality worldwide (1,2). Gastric cancer is characterized by a high mortality rate and a short median survival time, since it is too late for treatment when the diagnosis is made, in part due to the asymptomatic nature in the early stages of disease (3). According to the data from the 2008 World Health Organization (4) statistics, there were 988,000 new cases of gastric cancer worldwide and 736,000 mortalities, with ~50% of the cases occurring in China. Currently, gastric cancer remains a formidable disease to resolve, although great progress has been made in its treatment. Currently, the main therapeutic modalities involve surgery, radiation and chemotherapy.

The induction of cell cycle arrest and apoptosis has been demonstrated to induce cancer cell death and may be considered as a strategy to deal with gastric cancer (5). Currently, the cell cycle has been investigated widely and the cyclin-dependent kinase 1 (CDK1)/cyclin B complex has been found to play a significant role in the regulation of the G_2_/M phase (6). Apoptosis is a form of cell death that can be activated through at least two signaling pathways, the caspase-dependent and caspase-independent pathways, and several involve the mitochondria and B-cell lymphoma-2 (Bcl-2) family proteins (7).

Cantharidin (CTD) is an effective component extracted from blister beetles and is one of numerous natural products used in traditional Chinese medicine (8,9). The molecular formula of CTD is C_10_H_12_O_4_ and the molecular weight is 196.2. CTD has been reported to possess antibiotic and antiviral activities (10). Also, it has been used as an abortifacient and as a treatment for edema and warts (10,11). Recently, CTD has been demonstrated to exhibit potent anticancer activities on numerous types of human cancer cells, including pancreatic (12), bladder (13), breast (14) and hepatocellular cancer (15). It has also been reported that the CTD-induced reduction in cell growth and cell death in COLO 205 cells is due to the induction of cell apoptosis, which is associated with the death receptor and mitochondrial apoptotic pathways (8). CTD has also been suggested to be a novel and potent multidrug resistance reversal agent and may be a possible adjunctive agent for chemotherapy (16). In addition, CTD has been revealed to be a potent inhibitor of PP2A, which may block anaphase-promoting complex activity (17,18).

However, it remains unclear whether CTD induces cell cycle arrest and cell apoptosis in gastric cancer cells. Therefore, the aim of the present study was to investigate the effect of CTD in human gastric cancer cells and to explore the underlying mechanisms of this effect.

## Materials and methods

### Chemicals, reagents and antibodies

CTD was purchased from Sigma-Aldrich (St. Louis, MO, USA) and high-performance liquid chromatography was used to confirm that the purity was >99%. CTD was dissolved in cell culture medium at a stock concentration of 100 mg/ml and stored at −20°C. The stock solution was freshly diluted in the medium immediately prior to use in each experiment. The primary antibodies for cyclins A and B, CDK1, Bcl-2, Bad and poly ADP ribose polymerase (PARP) were purchased from Abcam Inc. (Cambridge, UK). The p21-specific antibody was obtained from Santa Cruz Biotechnology (Santa Cruz, CA, USA) and the antibodies for caspases-3, -7, -8 and -9 and Bid were obtained from Cell Signaling Technology (Beverly, MA, USA). β-actin was purchased from Santa Cruz Biotechnology. Horseradish peroxidase (HRP)-conjugated secondary goat anti-mouse and goat anti-rabbit secondary antibodies were purchased from Pierce. MTS was obtained from Promega (Madison, WI, USA).

### Cells and cell culture

The human gastric cancer SGC-7901 and BGC-823 cell lines were obtained from the Cell Bank of the Shanghai Institute of Biochemistry and Cell Biology, Chinese Academy of Sciences (Shanghai, China), where they were tested and authenticated. These procedures included cross-species checks, DNA authentication and quarantine. All the cell lines used in the present study were in culture for less than six months, and were maintained in RPMI 1640 medium (Gibco, Grand Island, NY, USA), with a 10% fetal calf serum and 1% penicillin/streptomycin mixture at 37°C in a humidified atmosphere of 5% CO_2_ and 95% air.

### Cell proliferation analysis by MTS assay

MTS assays were employed to examine the viability of the human gastric cancer SGC-7901 and BGC-823 cells treated with CTD. In brief, the cells were seeded at a concentration of 4–8×10^3^ cells/well on 96-well plates, with a total volume of 200 μl medium, and then cultured for 24 h to allow for attachment. The cells were treated with various concentrations of CTD (0–80 μM) for various periods of time (0–72 h). Two hours prior to the end of incubation, MTS (20 μl in 100 μl RPMI-1640 medium) was added to each well and the cells were incubated for 2 h at 37°C. The results were obtained by measuring the absorbance of each well at 490 nm and the half maximal inhibitory concentration (IC_50_) values were calculated using probit analysis.

### Cell cycle analysis by flow cytometry

Human gastric cancer SGC-7901 and BGC-823 cells were seeded at a concentration of 10×10^5^ cells/well on 6-well plates and were incubated with a 0–20-μM graded concentration of CTD for 24 h. The cells were then harvested and washed by centrifugation. For the determination of the cell cycle, the cells were fixed with 70% ethanol at −20°C overnight, then the cell pellet was re-suspended in phosphate-buffered saline (PBS) containing 40 μg/ml propidium iodide (PI) and 100 μg/ml ribonuclease A, and was incubated in the dark at room temperature for 30 min. The cell cycle distributions were determined by flow cytometry (BD FACSCalibur^TM^; Becton-Dickinson, Franklin Lakes, NJ, USA).

### Apoptosis analysis by flow cytometry

An annexin V-fluorescein isothiocyanate (FITC)/PI double-fluorescence apoptosis detection kit (Kaiji Biotech, Nanjing, China) was employed to quantify the apoptosis of the human gastric cancer SGC-7901 and BGC-823 cells treated with CTD. In brief, the cells were exposed to CTD (0–80 μM) on 6-well plates (2×10^5^ cells/ml) for 24 h. Next, using the Annexin V-FITC/PI double-fluorescence apoptosis detection kit according to the manufacturer’s instructions, the cells were stained. The results were obtained by analyzing the samples using a FACSCalibur flow cytometer (BD Biosciences, San Jose, CA, USA) within 1 h post-staining.

### Western blot analysis

SGC-7901 and BGC-823 cells in the exponential growth phase were treated with a graded concentration (0, 20, 40 and 80 μM) of CTD for 24 h. The cells were harvested and proteins were extracted from the cells at a density of 1×10^5^ cells/ml for 30 min in a radioimmunoprecipitation assay buffer containing 50 mmol/l TrisHCl, 150 mmol/l NaCl, 2 mmol/l EDTA, 2 mmol/l ethylene glycol-bis(β-aminoethylether), 25 mmol/l NaF, 25 mmol/l β-glycerophosphate, 0.1 mmol/l Na orthovanadate, 5 mg/ml leupeptin, 0.1 mmol/l phenylmethylsulfonyl fluoride, 0.2% Triton-X100 and 0.3% NP-40. The lysed solution was centrifuged at 13,000 × g for 20 min at 4°C. The protein levels were quantified using a bicinchoninic acid protein assay kit (Pierce, Rockford, IL, USA), following the manufacturer’s instructions. Equivalent amounts of protein were separated by 10% sodium dodecyl sulfate-polyacrylamide gel electrophoresis and then transferred to polyvinylidine difluoride membranes (Millipore, Billerica, MA, USA). The membranes were blocked in PBS containing 5% w/v skimmed dry milk and incubated at 4°C overnight with antibodies against cyclins A and B, CDK1, p21, caspases-3, -7, -8 and -9, Bcl-2, Bad, Bid and PARP at the recommended dilution, and then were finally incubated with HRP-conjugated goat anti-mouse and goat anti-rabbit secondary antibodies at room temperature for 1 h using enhanced chemiluminescence reagents (Cell Signaling Technology) and exposed to X-ray film.

### Statistical analysis

All the data were obtained from at least three independent experiments. The results were expressed as the mean ± standard deviation. Statistically significant differences between the experimental and control groups were identified by one-way analysis of variance. The IC_50_ values and 95% confidence intervals were calculated from the MTS assay data by probit regression. All statistical analyses were performed using SPSS 16.0 software (SPSS, Inc., Chicago, IL, USA). The P-values were two-tailed and a P≤0.05 was considered to indicate a statistically significant difference.

## Results

### CTD inhibits proliferation in SGC-7901 and BGC-823 cells

**Figure 1:**
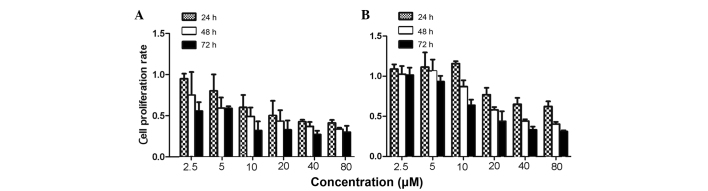
Cantharidin (CTD) affects proliferation in (A) SGC-7901 and (B) BGC-823 cells. The cells were treated with various concentrations of CTD (0, 2.5, 5, 10, 20, 40 and 80 μM) for various periods of time (24, 48 and 72 h), and MTS assays were used to examine the proliferation of the cells.

To evaluate the effects of CTD on the proliferation of human gastric cancer cells, MTS assays were used to measure the growth of SGC-7901 and BGC-823 cells. The present results revealed that CTD inhibited the proliferation of the SGC-7901 and BGC-823 cells in a dose- and time-dependent manner (Fig. 1). After 24 h, the IC_50_ value for CTD was 20.87±3.56 μM in the SGC-7901 cells, while after 48 and 72 h, the values were 12.14±1.28 and 6.45±2.10 μM in the SGC-7901 cells, and 30.25±0.48 and 18.90±2.51 μM in the BGC-823 cells, respectively.

### CTD induces G_2_/M phase arrest in SGC-7901 and BGC-823 cells

**Figure 2:**
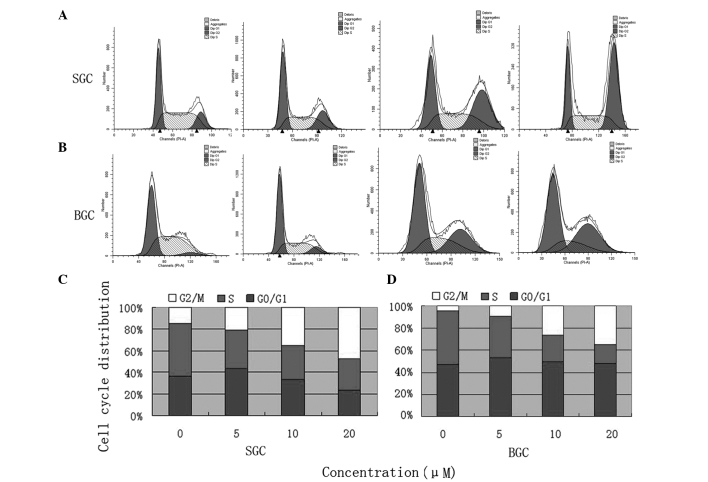
Cantharidin (CTD) affects cell cycle distribution in (A and C) SGC-7901 and (B and D) BGC-823 cells. The cells were treated with a graded concentration (0, 5, 10 and 20 μM) of CTD for suitable periods of time with 24-h intervals. Flow cytometry was used to analyze cell cycle progression.

To investigate whether CTD induced inhibition of SGC-7901 and BGC-823 cell growth via cell cycle-arresting mechanisms, flow cytometry was used to analyze cell cycle distribution subsequent to the cells being treated with 0, 5, 10 and 20 μM CTD for 24 h. In the SGC-7901 cells treated with 0, 5, 10 or 20 μM CTD, the ratios of cells in the G_2_/M phase were 14.67, 20.71, 34.92 and 47.32%, respectively. The ratios for the BGC-823 cells treated with 0, 5, 10 and 20 μM of CTD were 4.31, 9.66, 26.12 and 34.97%, respectively. The results revealed that CTD increased the ratio of cells in the G_2_/M phase in the SGC-7901 and BGC-823 cells in a dose-dependent manner (Fig. 2).

### CTD induces apoptosis in SGC-7901 and BGC-823 cells

**Figure 3:**
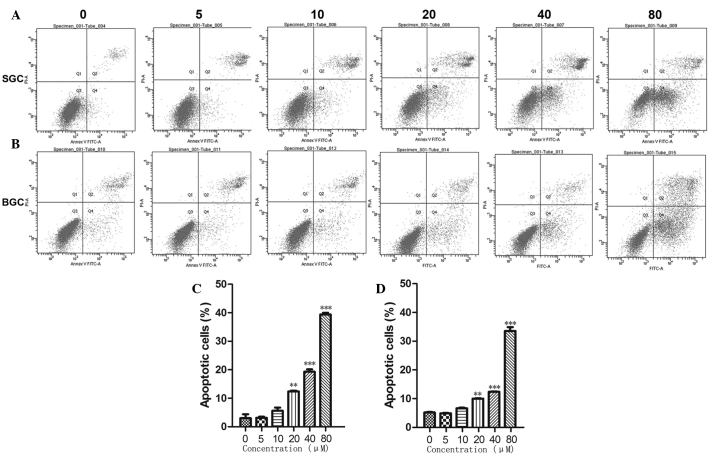
Cantharidin (CTD) affects cell apoptosis in (A and C) SGC-7901 and (B and D) BGC-823 cells. The cells were treated with a graded concentration of 0, 5, 10, 20, 40 or 80 μM of CTD for 24 h, and flow cytometry was used to analyze cell apoptosis. The results were analyzed by one-way analysis of variance. ^**^P<0.01 and ^***^P<0.001.

To investigate whether CTD induced inhibition of SGC-7901 and BGC-823 cell growth via the cell apoptotic pathways, cell apoptosis was measured by flow cytometry. As shown in Fig. 3, the cells treated with a high concentration of CTD exhibited much higher rates of apoptosis compared with the cells treated with a low concentration. The results revealed that CTD induced the apoptosis of the SGC-7901 and BGC-823 cells in a dose-dependent manner.

### CTD regulates the expression of G_2_/M phase- and apoptosis-associated proteins in SGC-7901 and BGC-823 cells

**Figure 4:**
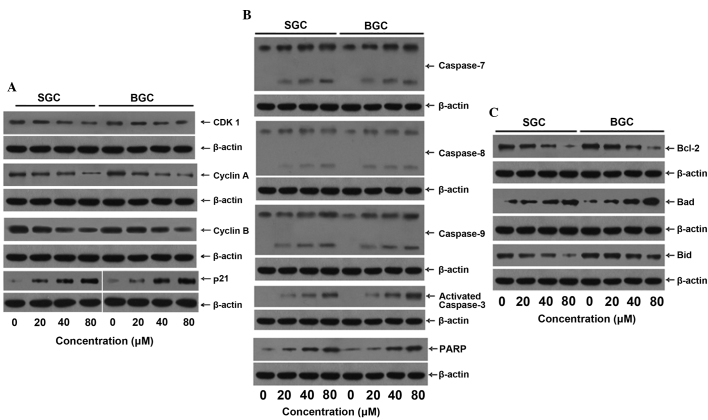
Cantharidin (CTD) affects the expression of (A) G_2_/M phase-associated and (B) apoptosis-associated proteins in SGC-7901 and BGC-823 cells. The cells were treated with 0, 20, 40, 80 μM CTD for suitable periods of time with 24 h intervals, and western blot analysis was used to determine protein expression.

The possible signaling pathways through which CTD induces cell cycle arrest and apoptosis in SGC-7901 and BGC-823 cells were investigated. Western blot analysis revealed that the protein expression levels of CDK1 and cyclins A and B were decreased following exposure to CTD in the two cell lines, but that the level of p21 was markedly increased (Fig. 4A), which led to cell cycle arrest and cell apoptosis. CTD increased the level of caspases-7, -8 and -9, activated caspase-3, PARP and Bad, but decreased the level of Bcl-2 and Bid (Fig. 4B).

## Discussion

In China, gastric cancer is one of the most common types of cancer and gastric cancer-related mortality accounts for ~23% of all cancer-related mortalities (19). It is therefore vital to solve such a formidable problem. Chemotherapy is becoming one of the main therapeutic modalities for the treatment of gastric cancer. The search for a new drug or therapeutic method is important, since the current treatments for gastric cancer are not ideal. Certain traditional Chinese medicines, including CTD, have been demonstrated to be reasonable and effective treatments for cancer. It has been reported that CTD has an effect on several types of human cancer, including pancreatic (12), bladder (13), hepatocellular (15) and colorectal (20) cancer. However, it remains unclear whether CTD is effective in treating gastric cancer as well. Therefore, in the present study, the function of CTD in human gastric cancer cells was examined and the underlying mechanisms were investigated.

The present study revealed that CTD inhibited the proliferation of human gastric cancer SGC-7901 and BGC-823 cells *in vitro* in a dose-dependent and time-dependent manner. The SGC-7901 and BGC-823 cells that were treated with CTD revealed G_2_/M phase arrest in cell cycle distribution. In addition, CTD induced apoptosis in the SGC-7901 and BGC-823 cells in a dose-dependent manner. It was indicated that CTD may inhibit the proliferation of human gastric cancer cells by inducing G_2_/M phase arrest and cell apoptosis.

Distinct protein kinase complexes, including the cyclins that are necessary for the activity of CDC/CDK kinase, have been reported to control the cell cycle (21). Cyclin D/CDK2, 4, 5 or 6 play an important role in the regulation of the G_1_ phase, and the cyclin E/CDK2 and cyclin A/CDK2 complexes are associated with the G_1_/S and S phases, respectively. By contrast, cyclin B/CDK1 is pivotal for cell progression through the G_2_/M phase (6,22–24). The activity of CDK1 kinase first depends on the accumulation of the cyclin B content. The synthesis of cyclin B starts late in the G_1_ phase and its content continually increases during S phase until cyclin B levels reach their maximum level in the G_2_ phase. Once cyclin B levels increase to a certain degree, the activity of CDK1 kinase appears. The activity of CDK1 kinase also increases to a maximum level in late G_2_ phase and this level is maintained until the middle of M phase (25). p21, as a potent cyclin-dependent kinase inhibitor, is a pivotal regulator and can also inhibit the activity of CDK1 and strengthen G_2_/M phase arrest (8,26,27). The G_2_/M checkpoint presents a potential target in the cell cycle for cancer therapy (28). Cyclin A also plays an important role in the initiation process of the S phase (25).

The present results revealed that CTD decreased the protein levels of CDK1 and cyclin B and increased the level of p21 in the SGC-7901 and BGC-823 cells, suggesting that CTD may induce G_2_/M phase arrest through downregulation of cyclin B and CDK1 and upregulation of p21. This finding is similar to previous studies in which CTD was revealed to induce G_2_/M phase arrest in the human bladder cancer T24 cell line and in pancreatic cancer cells (12,29), and to induce G_0_/G_1_ phase arrest in human bladder cancer TSGH 8301 cells (13). These findings require further investigation. Additionally, in the present study, the observation of a decreased level of cyclin A in the SGC-7901 and BGC-823 cells led to the suggestion that CTD may inhibit the process of S phase by downregulating cyclin A.

Apoptosis plays a central role in anti-tumorigenesis and involves two main signaling pathways, the death-receptor extrinsic pathway and the mitochondrial intrinsic pathway (30). These pathways are triggered by caspase-8 and -9, respectively. Membrane-associated protein complexes activate procaspase-8, while procaspase-9 is activated by mitochondria-associated protein (31). In addition, the two pathways converge at the point of caspase-3 activation. Following the activation of caspase-3, certain specific substrates for caspase-3, including PARP proteins, are cleaved, which is important for the development of apoptosis (32). The present results revealed that CTD increased the number of apoptotic cells and the level of caspase-7, -8 and -9, activated caspase-3 and PARP in the SGC-7901 and BGC-823 cells, suggesting that CTD may induce apoptosis via activation of a caspase cascade. In addition, Bcl-2 family proteins play a crucial role in regulating cell life. Three subfamilies of Bcl-2 family proteins have been identified in the apoptotic response, including the Bcl-2 subfamily, which includes Bcl-2 and Bcl-XL. The Bcl-2 subfamily is associated with the inhibition of apoptosis, whereas the Bax subfamily, which includes Bax, Bak and Bcl-Xs, and the BH3-only subfamily, which includes Bid and Bad, each promote apoptosis (33). The present study found that CTD upregulated Bad and downregulated Bcl-2 and Bid, suggesting that CTD may induce the apoptosis of SGC-7901 and BGC-823 cells by regulating Bcl-2 family proteins. Previous studies revealed that CTD induced apoptosis through the death receptor and mitochondrial apoptotic pathways in COLO 205 cells, while apoptosis was mediated through the JAK/STAT pathway in myeloma cells and through mitochondria-dependent signal pathways in human bladder cancer TSGH 8301 cells (8,13,34). It was indicated that the pathways may vary between cell lines. However, the tissue specificity of CTD requires further investigation.

In conclusion, the present results indicate that CTD may inhibit the proliferation of human gastric cancer SGC-7901 and BGC-823 cells *in vitro* by inducing G_2_/M phase arrest and cell apoptosis. CTD may induce G_2_/M phase cell-cycle arrest by downregulating cyclin A and B and CDK1, and upregulating p21. Apoptosis may be induced by CTD by the activation of a caspase cascade through the upregulation of caspases-7, -8 and -9, activated caspase-3 and PARP. In addition, CTD regulates Bcl-2 family proteins through the upregulation of Bad and the downregulation of Bcl-2 and Bid, which may also induce apoptosis.
